# Mechanical Properties of Twisted Carbon Nanotube Bundles with Carbon Linkers from Molecular Dynamics Simulations

**DOI:** 10.3390/ijms24032473

**Published:** 2023-01-27

**Authors:** Andrea Pedrielli, Maurizio Dapor, Konstantinos Gkagkas, Simone Taioli, Nicola Maria Pugno

**Affiliations:** 1 Fondazione Bruno Kessler, Via Sommarive 18, Povo, 38123 Trento, Italy; 2 European Centre for Theoretical Studies in Nuclear Physics and Related Areas (ECT*), Fondazione Bruno Kessler, Strada delle Tabarelle 286, Villazzano, 38123 Trento, Italy; 3 Trento Institute for Fundamental Physics and Applications (TIFPA-INFN), Via Sommarive 14, Povo, 38123 Trento, Italy; 4 Advanced Material Research Division, Toyota Motor Europe NV/SA, Hoge Wei 33B, 1930 Zaventem, Belgium; 5 Faculty of Applied Physics and Mathematics, Gdańsk University of Technology, 11/12 Gabriela Narutowicza Street, 80-233 Gdańsk, Poland; 6 Laboratory for Bioinspired, Bionic, Nano, Meta Materials & Mechanics, Department of Civil, Environmental and Mechanical Engineering, University of Trento, Via Mesiano 77, 38123 Trento, Italy; 7 School of Engineering and Materials Science, Queen Mary University of London, Mile End Road, London E1 4NS, UK

**Keywords:** nanotube bundles, mechanical properties, carbon linkers, force fields, molecular dynamics

## Abstract

The manufacturing of high-modulus, high-strength fibers is of paramount importance for real-world, high-end applications. In this respect, carbon nanotubes represent the ideal candidates for realizing such fibers. However, their remarkable mechanical performance is difficult to bring up to the macroscale, due to the low load transfer within the fiber. A strategy to increase such load transfer is the introduction of chemical linkers connecting the units, which can be obtained, for example, using carbon ion-beam irradiation. In this work, we investigate, via molecular dynamics simulations, the mechanical properties of twisted nanotube bundles in which the linkers are composed of interstitial single carbon atoms. We find a significant interplay between the twist and the percentage of linkers. Finally, we evaluate the suitability of two different force fields for the description of these systems: the dihedral-angle-corrected registry-dependent potential, which we couple for non-bonded interaction with either the AIREBO potential or the screened potential ReboScr2. We show that both of these potentials show some shortcomings in the investigation of the mechanical properties of bundles with carbon linkers.

## 1. Introduction

Lightweight fibers characterized by high modulus and strength can have a disruptive impact on applications ranging from bulletproof tissues to aeronautics and space technologies. To deliver high-performance fibers for such high-end applications, carbon-based materials [1,2,3,4,5,6], and in particular carbon nanotubes (CNTs), are the most natural and promising candidates. In this respect, the challenge is to scale up while keeping the CNTs extremely high in strength and stiffness (e.g., 100 GPa and 1 TPa for the (5,5) CNTs [7]) to the macroscale. Indeed, in order to be implemented in real-world applications, fibers composed of a large number of aligned CNTs are needed.

Recently, large improvements have been claimed in the preparation of CNT bundles [8], reaching 80 GPa of tensile strength with ultra-long CNT-based fibers several centimeters long. These results were achieved by releasing the non-uniform initial strains of the CNTs in the bundle. Defects, such as vacancies, non-hexagonal rings, and interstitial defects, of course, decrease the mechanical properties of carbon nanotubes. However, when the CNTs are shorter than the whole fiber, the latter turns out to be far weaker than its constituents [9,10,11,12,13]. Thus, we believe that the presence of these defects is not crucial in determining the mechanical properties of the fiber, as the limiting factor is given by the load transfer between the CNTs. We notice that the influence of defects on the mechanical properties of the fibers becomes relevant only if the load transfer between the CNTs approaches their intrinsic resistance.

In general, the failure of CNT fibers can be due either to the slipping of the nanotubes within the bundles or to the failure of a single nanotube, or both. Concerning the former aspect, we notice that the adhesion between bare CNTs is driven by weak van der Waals (vdW) interactions. The latter could be enhanced by using, for example, bundles of collapsed CNTs or twisted yarns [14,15]. The production of such yarns can be obtained e.g., through a solid spinning process starting from super-aligned CNT forests [16,17]. A different strategy relies on the interposition of linkers between the CNTs [18,19] or the induction of defects and chemical bonds by means of electron beams [20,21,22], noble gases [21,23], or carbon ions [24,25].

Methods based on chemical cross-linking produced through irradiation rely on the positive balance between the increase in the load transfer due to cross-linkers and the worsening of the intrinsic mechanical properties of the nanotubes owing to the formation of defects. In principle, both electron beams or ions could be used to induce defects. However, irradiation via carbon ions is the most promising approach, as it creates interstitial sites while avoiding the introduction of substitutional impurities into the system. A particularly interesting situation is when carbon atoms, occupying interstitial positions, act as cross-linkers that are crucial to prevent sliding between nanotubes [25]. We note that possible adatoms can easily migrate towards interstitial positions between the nanotubes [26,27]. This case, with single carbon atom linkers, will be assessed in the present work.

Computer simulations [19] have shown that a small percentage of linkers is sufficient to strongly increase intertube load transfer and to produce defective points in the nanotubes, such as distortion and vacancies. Chemical cross-linking is among the most promising strategies for the full exploitation of the outstanding properties of CNTs. Furthermore, the interposition of the linkers and twisting have been recently coupled together [28], while the load transfer for non-twisted fibers has been increased using adhesion agents [29]. In this respect, mesoscale models have been developed to bridge the gap between atomistic simulations and experimental scales [30].

In this work, we investigate, by means of molecular dynamics (MD) simulations, the (high-velocity) mechanical properties of twisted CNT bundles covalently connected by single-carbon linkers [24,25]. The collective interaction of the nanotubes within the bundle is evaluated by assessing the stress–strain curves.

Several theoretical and computational studies have been devoted to studying the mechanical properties of twisted nanotubes [31,32], twisted yarns [15], and to the search for the best strategies aimed at the improvement of the mechanical properties of CNT bundles [15,19,33,34,35,36,37,38,39,40]. Nevertheless, the combined effect on such mechanical properties of coupling the fibers using interstitial carbon atom linkers together with the twisting of the nanotube bundles is still unexplored.

Here, we focus on CNT bundles that may be of technological interest and that can be manufactured using current techniques based on carbon-ion beam irradiation. In particular, we center our discussion on bundles of seven (6,6) nanotubes. This choice is driven from one side by the higher mechanical properties of thin nanotubes, and from the other side by the symmetry of the structure that allows for easy insertion of the linkers. Furthermore, for small bundles, the distribution of linkers from carbon ion irradiation is more uniform than for larger bundles. These small bundles can be used to compose larger yarns. The main limitation of our model is the length of the finite-length nanotubes, which is 20 nm; longer nanotubes would clearly be more representative of the experimental realization of such bundles. However, the computational cost of the simulation scales proportionally with the length of the nanotubes. Thus, to demonstrate the efficacy of the proposed strategies for increasing the mechanical strength of CNT bundles, we start studying these systems using an affordable computational model with a limited number of carbon atoms and leave to further investigations CNTs with structural characteristics similar to the experimental setup.

In order to investigate the mechanical properties of materials using molecular dynamics, a reliable model of interatomic interactions is needed. With respect to CNTs, many different force fields have been developed. Among them, the AIREBO [41] is a widely used and reliable potential to determine the mechanical properties of carbon-based materials [42,43]. A standard AIREBO is known to overestimate the mechanical properties of these materials, in particular in the near-fracture regime. A strategy to overcome this shortcoming is a change in the internal cutoff (AIREBO-mod) to obtain better fracture behavior [44]. We rely on this potential for the description of our systems.

Nevertheless, recently some improvements have been achieved in the development of accurate force fields for carbon. ReboScr2 [45] interatomic potential has been developed for the description of bond breaking. Furthermore, the dihedral-angle-corrected registry-dependent interlayer potential (DRIP) [46] force field for the description of interlayer (or intertube) interactions has been proposed. This force field can be coupled with a potential in dealing with bonded interactions, such as REBO [47] or Tersoff [48].

ReboScr2 has been used to investigate the structural properties of CNT bundles with linkers induced via irradiation [49]. In particular, using the ReboScr2 potential, the authors analyzed bond types, defects, and the optimal distribution of linkers. However, the mechanical properties of these structures were not assessed.

We discuss the limitation of these novel potentials using the results of pull-out simulations. From our results, we conclude that these novel potentials still show some shortcomings in the description of the stress–strain regimes, in which an accurate description of the vdW forces and of the bonded interactions is fundamental, as well as that of bond-breaking during the fracture of CNT bundles.

## 2. Materials and Methods

Our investigation focused on three systems:

a Finite-length bundles with or without linkers made by bare CNTs for the evaluation of the intertube interaction. We also used these samples for the assessment of the ReboScr2 and DRIP potentials. For this first type of system, we performed pull-out tests. The samples were set up in 20 nm-length bundles composed of seven (6,6) CNTs.
b Continuous straight and twisted CNT bundles with or without linkers. In these systems, the periodicity was added to the seven (6,6) CNT bundles via a periodic supercell with a longitudinal dimension of 20 nm. The model represents the physical case of a bundle composed of extra-long nanotubes.
c Periodic bundles composed of finite-length CNT bundles with or without linkers for the evaluation of linkers–twisting coupling. For these bundles composed of finite-length (6,6) CNTs, we shifted the outer CNT of the bundle, alternatively, up and down for 5 nm, then set up a periodic cell of 20 nm. The model represents the physical case of a long bundle composed of short nanotubes.

**Figure 1:**
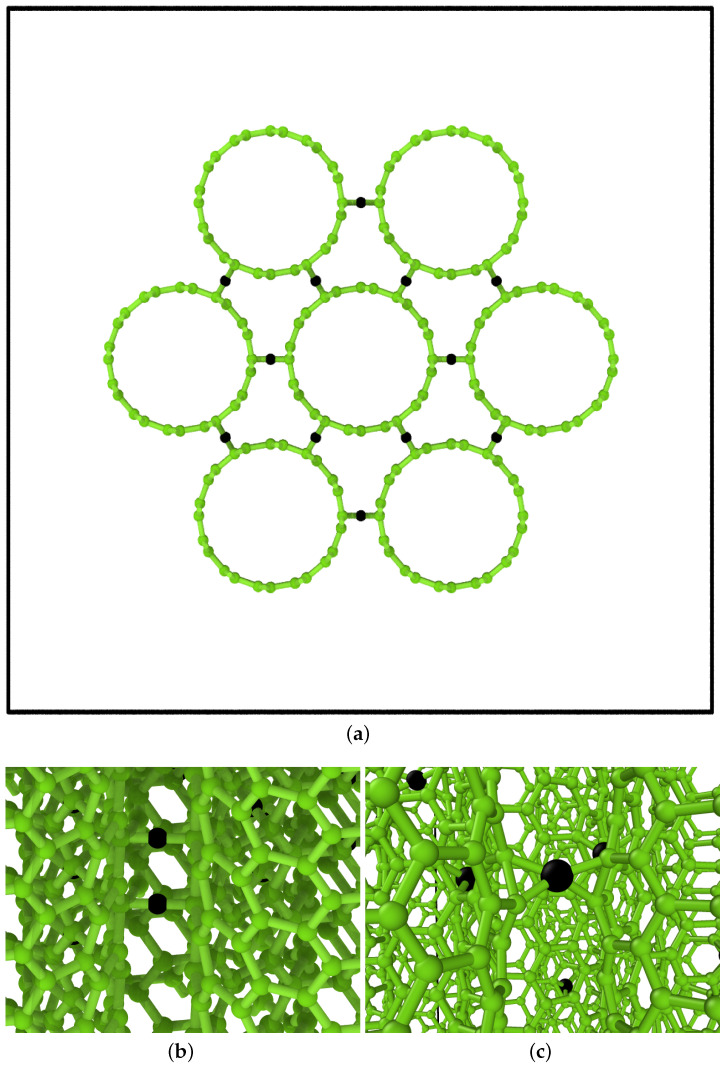
Computational model of the (6,6) CNT bundle (**a**), showing the hexagonal symmetry of the structure; the symmetry was exploited to insert the linkers (rendered in black). The linkers are covalently bonded with the nanotubes (**b**). Upon relaxation, they form tetravalent bonds (**c**).

The dangling bonds at the CNT edges were saturated using hydrogen atoms in the computational systems with finite-size nanotubes (systems (a) and (c)). To insert the carbon atom linkers within the bundle, we relied on the symmetry of the (6,6) CNTs as shown in Figure 1. The linkers were inserted with a random distribution in both the angular and longitudinal directions in the sites connecting the nearest neighbors of different nanotubes. While the linkers started in a symmetrically perfect position, upon relaxation of the atomic positions, the carbons formed tetravalent bonds with the adjacent sites. The percentage of linkers was calculated with respect to the number of atoms of the CNT bundle.

**Figure 2:**
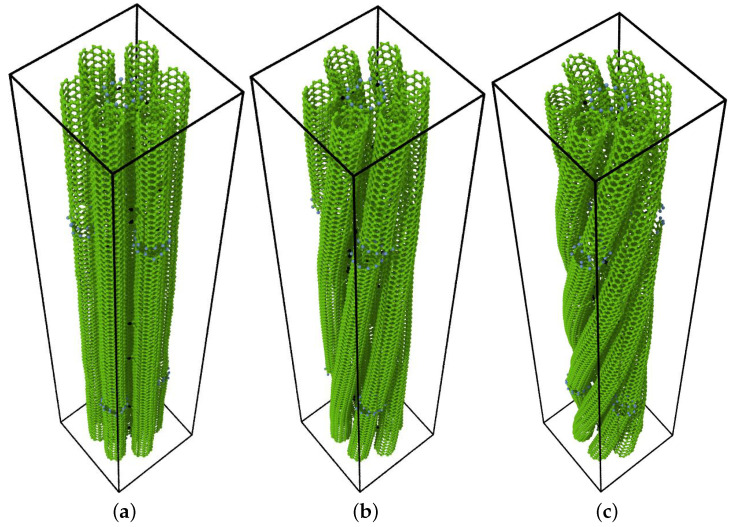
Structural models used for the simulations of the straight (**a**) and twisted (**b**,**c**) (6,6) CNT bundles with linkers connecting the nanotubes. The linkers are reported in black and are made of carbon atoms bonded to the nanotubes. Hydrogen atoms (in blue) saturate dangling bonds.

In systems (b) and (c) of Figure 2, we also considered the twisting of the samples. As shown in Figure 2, for the bundles composed of finite-length nanotubes (system (c)), the twisted bundles were prepared by twisting them in a rotation of 60° and 120° over a distance of 20 nm, which corresponds to a twist of 3°/nm and 6°/nm. Choosing these angles allows one to obtain periodic bundles while maintaining the length of the single nanotube at 20 nm.

In our set-up, the carbon linkers were inserted before the twist of the bundle, corresponding to the experimental condition of ion irradiation before the twist. A complementary study should investigate the effect of the linkers on bundles twisted before their insertion.

The mechanical properties were computed using molecular dynamics implemented using the LAMMPS code [50]. The calculations were performed using the AIREBO-mod [41,44], setting the internal cutoff to 2.0 Å. For the evaluation of their suitability in describing our systems, we used a DRIP [46] interaction coupled with the intralayer part of the AIREBO-mod [41,44] and ReboScr2 [45].

The AIREBO potential consists of three terms:

(1) $$E=\frac{1}{2}\sum_{i}\sum_{j\neq i}\left[E_{ij}^{\mathrm{REBO}}+E_{ij}^{\mathrm{LJ}}+\sum_{k\neq i,j}\sum_{l\neq i,j,k}E_{kijl}^{\mathrm{TORSION}}\right],$$

where *i*, *jk*, and *l* label the atoms. The first term is the reactive empirical bond order (REBO) potential term [47]; the second one is the Lennard-Jones (LJ) term for the non-bonded interaction; and the third is an explicit 4-body potential for the description of dihedral angles. The DRIP potential can replace the LJ part of the AIREBO.

The Langevin thermostat with a damping coefficient of 0.1 ps${}^{-1}$ to maintain a constant temperature T=300 K during the simulation was used. The pressure control was realized by means of the Nosé–Hoover barostat-thermostat [51]. The velocity Verlet [52] integrator with a time step of 0.5 fs allowed proper integration of Newton’s equations of motion. All the samples were fully equilibrated before the mechanical tests.

We imposed high-velocity stimuli on the samples, with a velocity typical of ballistic impact. Low-velocity stimuli can be studied using quasi-static simulations. The pull-out simulations—systems of type (a)—were performed by imposing a constant velocity displacement of 1.0 Å/ps on the top of the central nanotube and by computing the total stress on the system and, in particular, the component of the total stress along the pull direction. The bottom edge of the outer nanotubes was fixed along the axial direction while allowing lateral displacement.

The tension simulations (systems of type (b) and (c)) were performed by expanding the periodic simulation cell at a constant velocity of 1.0 Å/ps along the longitudinal direction and remapping the atoms in the longitudinal direction at each expansion step. We computed the total stress along the tension direction.

The strain parallel to the direction of deformation is defined by:

(2) $$\varepsilon =\frac{L-L_{0}}{L}=\frac{\Delta L}{L},$$

where $L_{0}$ and *L* are the initial and actual length of the sample in the direction of loading, respectively.

To determine the stress, the pressure stress tensor components in response to the external deformation are computed as [53]

(3) $$P_{ij}=\frac{\sum_{k}^{N}m_{k}v_{k_{i}}v_{k_{j}}}{V}+\frac{\sum_{k}^{N}r_{k_{i}}f_{k_{j}}}{V},$$

where *i* and *j* label the coordinates *x*, *y*, and *z*; *k* runs over the atoms; $m_{k}$ and $v_{k}$ are the mass and velocity of *k*-th atom; $r_{k_{i}}$ is the position of *k*-th atom; $f_{k_{j}}$ is the *j*-th component of the total force on the *k*-th atom due to the other atoms; and, finally, *V* is the volume of the simulation box.

We notice that the stress is normalized with respect to the annular cross-section of the single nanotube in the case of pull-out, while in the tests carried out on the whole CNT bundle, with respect to the sum of the annular sections of all the CNTs. The value of the radius for the central nanotube in the (6,6) bundles was 4.05 Å, while the conventional thickness was 3.35 Å. Images of atomic configurations were produced using the OVITO package [54].

## 3. Results

### 3.1. Pull-Out Test from a Nanotube Bundle

Previous studies on the mechanical properties of CNTs [19,49] have shown that a specific ratio of covalent carbon linkers over the total number of atoms of the bundle may change the mechanical failure from brittle to plastic. In particular, the two regimes correspond to the fracture of the CNTs within the bundle and to the fracture of the bonds with the subsequent sliding of the CNTs in the bundle, respectively.

**Figure 3:**
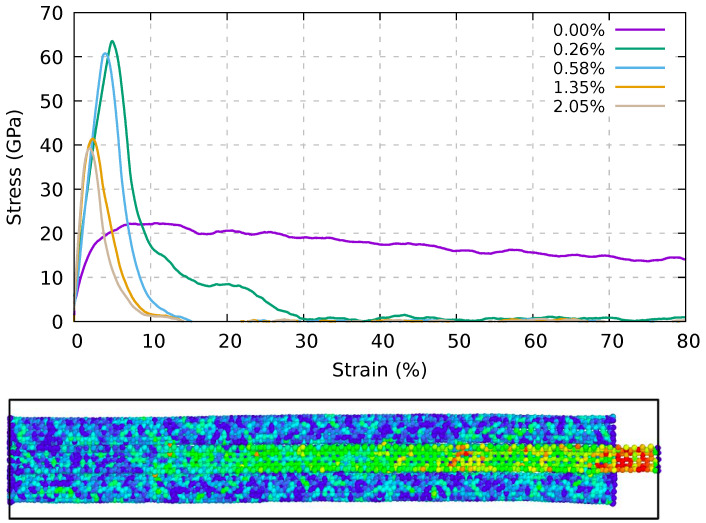
Top panel: pull-out stress for the central nanotube from the seven (6,6) CNT bundles with different percentages of linkers at a constant pull-out velocity of 1.0 Å/ps. Bottom panel: cross section of the bundle with a linker percentage of 0.26%; the color coding shows the von Mises stress distribution that decreases as the distance from the pull-out extremity increases.

We note that in [19], the authors used the plain AIREBO potential, which is known to cause an unphysical hardening under tension whenever the pull-out results in bond breaking. Nevertheless, this hardening may be strongly suppressed at a very low strain rate [55]. In Figure 3 we report the pull-out stress for different percentages of linkers, finding a brittle behavior for the lowest value. This percentage is similar to that found previously (0.226% [49]), although larger systems were used therein. In our simulations at a lower percentage of linkers, the plastic behavior is given by the sliding of the linkers between the two nanotubes, without causing their fracture. The four starting covalent bonds are maintained whilst the atoms change at which the linker bonds. At a large percentage of linkers, the behavior starts becoming brittle and the stress is progressively concentrated at the top of the central nanotube. Moreover, the stress along the central nanotube is not uniform and decreases as the distance from the pulled-out extremity increases. For that reason, while the modulus increases with the linker percentage, the fracture strain as well as the strength at maximum strain decreases. A longitudinal cross-section of the bundle with a linker percentage of 0.26% is reported in Figure 3 (bottom panel). The color coding shows the von Mises stress distribution that decreases as the distance from the pull-out extremity increases.

### 3.2. Twisted Bundles Composed of Extra-Long Nanotubes under Tension

The effect of twisting on the mechanical properties of nanotube bundles with carbon linkers is still to be investigated. In order to get insights into these linked systems, we model an infinite bundle using seven (6,6) CNT bundles with a periodic cell of 20 nm in length. The samples were initialized either straight or by imposing a linear rotation of 60° and 120° along the bundle axis, respectively. The bundles were tested by imposing a deformation with a constant velocity of 1.0 Å/ps.

For the twisting angles analyzed here, there is no collapse of the CNTs, showing only a small radial deformation. This can be attributed to the small diameter of the CNTs.

**Figure 4:**
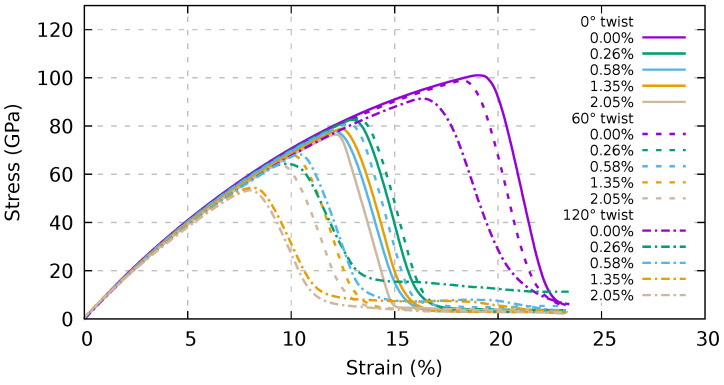
Dependence of the stress–strain response on twisting and on the percentage of linkers for an infinite bundle composed of (6,6) CNTs with a periodic supercell length of 20 nm. AIREBO-mod potential and a constant strain velocity equal to 1.0 Å/ps were used.

**Figure 5:**
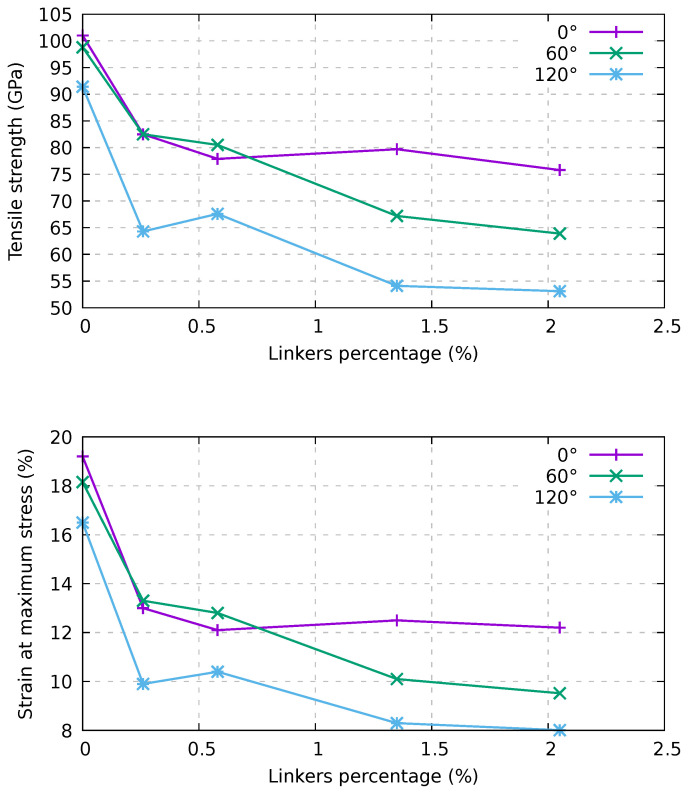
Dependence of the tensile strength (**top**) and of the strain at maximum stress (**bottom**) on twisting and percentage of linkers for an infinite bundle composed of (6,6) CNTs characterized by a periodic supercell of 20 nm in length. AIREBO-mod potential and a strain velocity of 1.0 Å/ps were used.

We report in Figure 4 the stress–strain curves of such bundles. It can be seen that the presence of both twisting and carbon linkers decreases the tensile strength and strain at the maximum stress of the bundle. In order to better investigate these effects, we present in Figure 5 the tensile strength and the strain at maximum stress, respectively, as functions of the twisting angle and of the percentage of linkers.

We focus on the tensile strength and strain at maximum stress, as they appreciably depend on both the linker percentage and the twisting angle. At odds, Young’s modulus in the limit of zero strain (see Figure 4) in the considered cases is essentially independent of those parameters. Indeed, one can note that the stress-strain curves collapse to a single curve in the limit of zero strain.

With regard to tensile strength, in the top panel of Figure 5, we show that for a low percentage of linkers (<1%), the straight and 60° twisted samples have very similar behavior, while the 120° twisted bundle presents a significant decrease in tensile strength. We also conclude that the tensile strength is almost constant beyond 1% of linkers in the straight sample, while it monotonically decreases in the twisted samples. At the percentage of linkers of 2%, the tensile strength is linear in the twisting angle. At maximum stress (bottom panel of Figure 5), the scenario is essentially the same, with a threshold at 1% of linkers, beyond which the 60° twisted sample properties diverge from those of the straight one. It can be worth noting that, for the 60° twisted sample at a low percentage of linkers of 0.58%, the mechanical characteristics seem to increase with respect to the straight bundle with the same percentage of linkers.

### 3.3. Twisted Bundles Composed of Short Nanotubes under Tension

**Figure 6:**
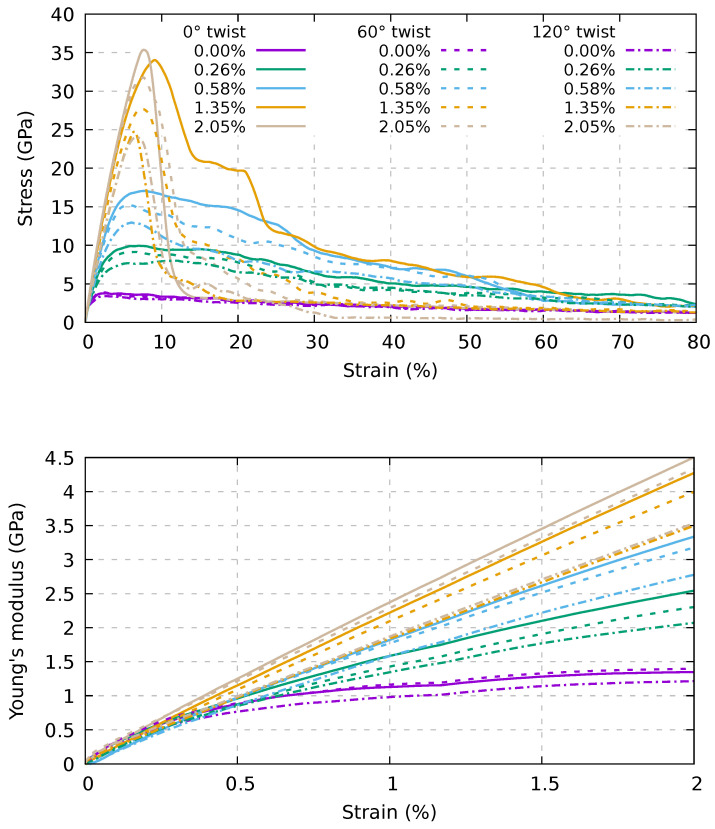
Dependence on twisting and percentage of linkers of the stress–strain response of the bundle composed of 20 nm (6,6) CNTs (**top**). In the **bottom** panel, a zoom of the low-strain part of the stress–strain curves reported in the top panel is shown. AIREBO-mod potential and a strain velocity of 1.0 Å/ps were used.

To approach the case where the constitutive elements of the bundles have a finite length, we used the models reported in Figure 2. We report in Figure 6 the stress–strain curves for the three samples, assessed using the AIREBO-mod potential model. It can be seen that there is a plastic-to-brittle transition between 0.58% and 1.35% of the percentage of linkers. The difference with the pull-out case can be rationalized by noting that the superposition of the CNTs within the bundle is shorter than for the pull-out, due to the shift of the outer nanotubes.

**Figure 7:**
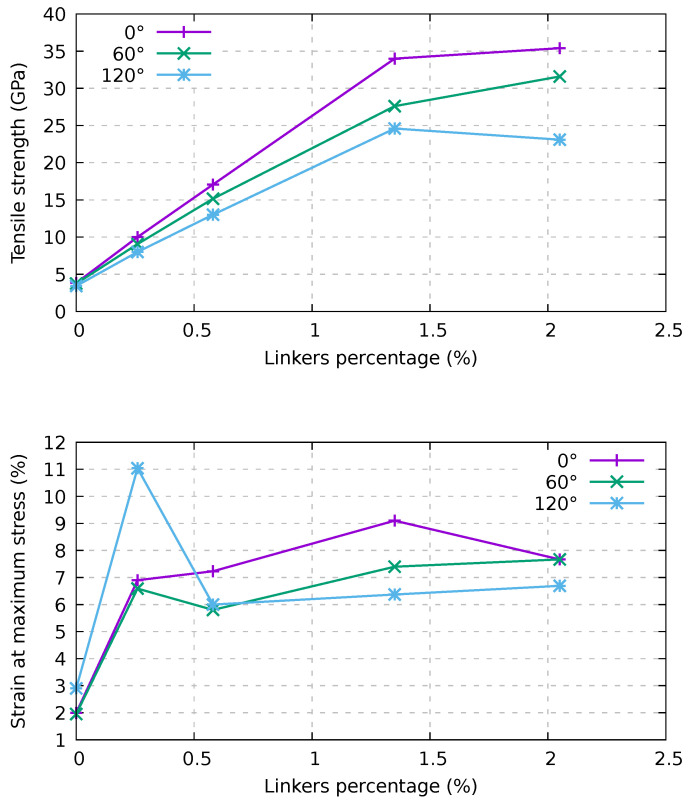
Dependence on twisting and percentage of linkers of the tensile strength (**top**) and strain at maximum stress (**bottom**) for an infinite bundle composed of (6,6) CNTs with a periodic supercell of 20 nm in length. AIREBO-mod potential and a strain velocity of 1.0 Å/ps were used.

The tensile strength and the strain at maximum stress are reported in the top and bottom panels of Figure 7, respectively, as a function of the twisting angle and of the percentage of linkers. In particular, in the top panel of Figure 7, we show that the increase in tensile strength is rather linear at a low percentage of linkers (up to about 1.4%), while beyond that value we find a saturation. The strain at maximum stress reported in the bottom panel of Figure 7 tends to be similar at all the percentages of linkers in the range of 6–10%.

**Figure 8:**
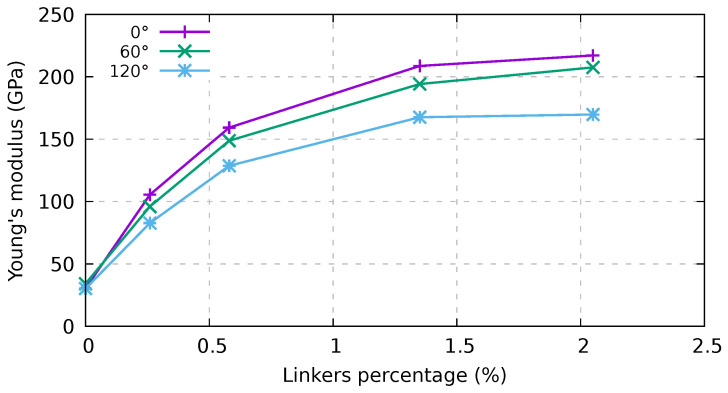
Dependence on twisting and percentage of linkers of Young’s modulus for an infinite bundle composed of (6,6) CNTs with a periodic supercell of 20 nm in length. AIREBO-mod potential and a strain velocity of 1.0 Å/ps were used.

Again, Young’s modulus (see Figure 6) is still independent of those parameters. This behavior is expected for bundles composed of infinite length CNTs; for finite length CNTs, this behavior is related to the high velocity of our test coupled with the remapping of the atoms. Indeed, the atoms are remapped in the cell during the simulations so that, in the first part of the stress–strain curve, Young’s modulus depends on the carbon atoms within the tubes rather than the linkers. After a short period of time, the stress within the bundles evolves differently for different structures. This interpretation is supported by the fact that, for the samples with the higher percentage of linkers, the slope of the stress–strain curve is almost constant up to 2% strain. Moreover, for infinite-length bundles, Young’s modulus is the same in all cases, and the curves are almost identical up to 5% strain; at odds, finite-length nanotubes start diverging after 0.25% strain. In the latter case, we can compute Young’s modulus using values between 0.25% and 2% strain, where they are rather linear. Young’s modulus values for the various samples are reported in Figure 8. We note that Young’s modulus increases monotonically with an increase in linker percentage and decreases with the twisting angle. It is also worth noting the difference in tensile strength reported in Figure 7 (top panel), where the tensile strength increases linearly with the linker percentage (up to about 1.4%). Young’s modulus presents a large increment at small strain and then smoothly saturates beyond 1.4%. These findings suggest that an increment of Young’s modulus can be obtained at a lower linker percentage, while, conversely, to maximize such increment in the tensile strength, a higher linker percentage should be adopted.

**Figure 9:**
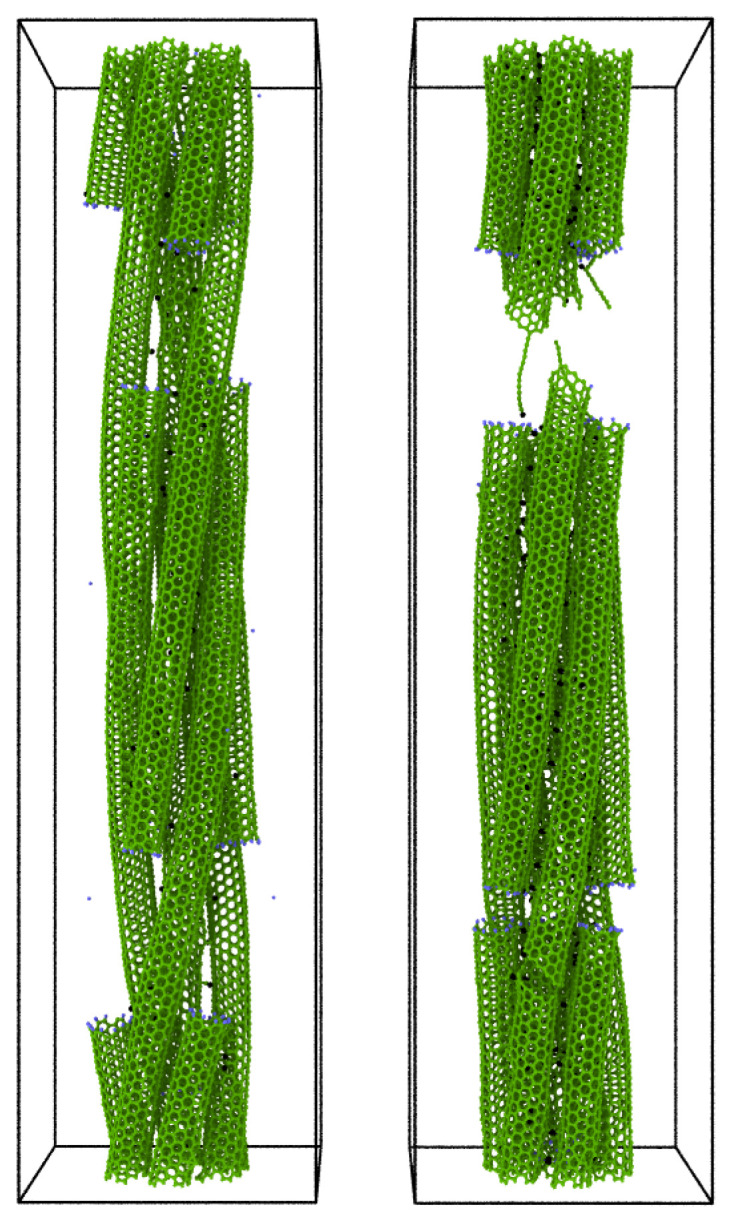
Snapshot at 15% strain of the 120° twisted bundle composed of 20 nm (6,6) CNTs for a percentage of linkers of 0.58% (**left**) and 1.53% (**right**). A different percentage of linkers corresponds to different failure mechanisms: the slipping of the nanotubes (**left**) and the fracture of the nanotubes (**right**), respectively.

We report in Figure 9 two snapshots at 15% strain of the 120° twisted bundle composed of 20 nm (6,6) CNTs, for a percentage of linkers of 0.58% (left) and 1.53% (right), respectively. These percentages of linkers correspond to different failure mechanisms: in the first case, the slipping of the nanotubes, and in the second, the fracture of the nanotubes.

### 3.4. Test of Novel Potentials by Means of Pull-Out Tests

Recently, the ReboScr2 [45] interatomic potential, which better describes the near-fracture regime dynamics by allowing the formation and breaking of chemical bonds, has been proposed [25,49,56,57,58,59]. ReboScr2 has also been used in the simulation of intertube coupling in CNT bundles to model the carbon ion irradiation process [25]. It should be noted that this screened potential describes correctly the interlayer distance in graphite, by including the vdW dispersion forces via a long-range interaction [45]. However, the description of the friction between different graphene layers or CNTs using ReboScr2 is not as accurate; here we show, in particular, that this potential overestimates the pull-out force of a CNT from a bundle.

On the other hand, the AIREBO potential does include the vdW interaction using an LJ form, so it is not able to correctly describe the registry dependence of the interaction. In fact, the interlayer interactions in graphene structures consist of both the vdW attractive force and the repulsive force due to the anisotropic overlap of electronic orbitals, which increases with sliding. As a result, the AIREBO strongly underestimates the energy variation of interlayer sliding with respect to higher-accuracy density functional theory (DFT) calculations [46], where the latter interaction is treated more accurately.

In this respect, a solution for the accurate description of the interlayer (or intertube) interactions, which accounts for both these contributions, is represented by the dihedral-angle-corrected registry-dependent interlayer potential (DRIP) [46]. DRIP, which includes the anisotropic (or registry-dependent) interaction, can be coupled with the potential that deals with bonded interactions, such as REBO, Tersoff [48], or the intralayer part of the AIREBO. Here, we test the DRIP potential by assessing the mechanical properties of CNT bundles. In particular, we compare the results obtained using DRIP coupled with the intralayer part of the AIREBO-mod (that is the AIREBO-mod in which the LJ interaction has been switched off) and with those obtained using the plain AIREBO-mod and ReboScr2 potentials.

The ReboScr2 potential [45] accounts for correct behavior in near and fracture regimes, and has been tested in several conditions, such as in the case of a graphene sheet under tensile load. Furthermore, it reproduces the correct interlayer distance in graphite. Here, we test a more complex case, where the sliding between CNTs, i.e., the pull-out of the central nanotube from a seven (6,6) CNT bundle, occurs.

**Figure 10:**
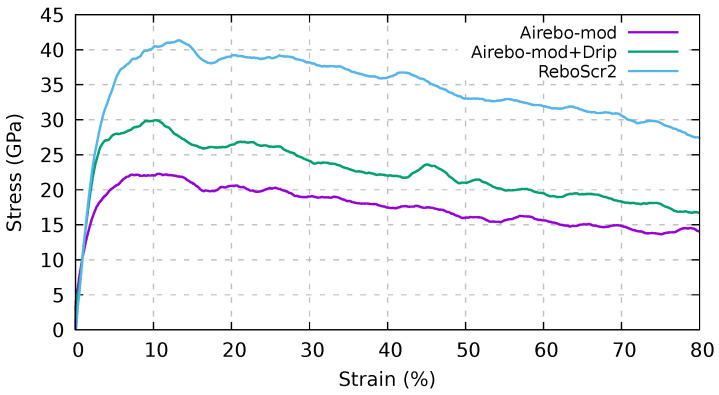
Pull-out stress for the central nanotube from the seven (6,6) CNT bundles with different potentials at a constant pull-out velocity of 1.0 Å/ps. The ReboScr2 potential overestimates the pull-out stress by a factor of two with respect to AIREBO-mod, and by 25% using the (AIREBO-mod+DRIP) interaction model.

In Figure 10 we show the pull-out stress for the three computational setups. In particular, the DRIP potential coupled with the AIREBO-mod represents our benchmark, in this case being the intertube interaction registry-dependent. With respect to the DRIP-AIREBO-mod, the ReboScr2 potential overestimates by one-third the pull-out stress, while the plain AIREBO-mod model potential (no DRIP) underestimates it by one-fourth. These results thus show a strong dependence on the potential used for carrying out molecular dynamics simulations of the sliding between bare CNTs without linkers. While it is preferable to use the DRIP-AIREBO-mod approach without linkers, in this work we stuck to AIREBO-mod potential, as it is more robust in the case of fracture of the samples and delivers near-fracture stress values similar (≃ 10 GPa) to the DRIP-AIREBO-mod potential.

## 4. Discussion

In this work, we investigated, by means of molecular dynamics simulations, the mechanical properties of twisted CNT bundles connected via covalent carbon linkers. In particular, we assessed the effect of the twisting angle and percentage of linkers in continuous bundles made by nanotubes characterized by infinite (extra-long) and 20 nm lengths. We assessed the collective interaction of the nanotubes within the bundle through the calculation of the stress–strain curves.

For the seven infinite-length (6,6) CNT bundles, we found a depletion of the mechanical properties, in particular with a linear decrease in tensile strength and in the strain at maximum stress with the twisting angle for a percentage of linkers beyond 1%. At a lower percentage of linkers, the effect of twisting is seen only for the 120° twisted sample. In the case of bundles made by 20 nm-long CNTs, the increase in tensile strength at a low percentage of linkers is linear up to ca. 1.4%, followed by saturation. The strain at maximum stress is instead almost independent of the linker percentage. For the range of linker percentages and twist angles considered here, the overall dependence of the above-mentioned mechanical properties on these two variables is approximately comparable, with the effect of linker percentage being two to three times larger than that attributed to twisting. However, aside from the case of infinite CNT bundles (see the upper panel of Figure 7), the mechanical performance cannot be explained by a simple superposition of the contribution given by the addition of linkers and of twisting. The interplay between linkers and twisting is actually not trivial. The mechanical properties have been assessed using the AIREBO-mod potential, as ReboScr2 seems unsuitable when friction plays a paramount role, such as in the case of bare nanotube bundles. We also compared the DRIP potential coupled with the intralayer AIREBO-mod interaction model with the plain AIREBO-mod, finding that the difference between the two models is up to 25% for bare nanotubes.

## 5. Conclusions

For CNT bundles, the interplay between the percentage of linkers and the twist angle was investigated using molecular dynamics simulations, finding significant interdependence of these two factors in affecting their mechanical properties. This work paves the way to further investigation of this interplay. Future computational studies should focus on increasing the length of the nanotubes and should also investigate different distributions of linkers, for example, determined via different chirality of the nanotubes. While the range of CNT lengths in the experimental setup can be very wide, spanning from tenths of nanometers to centimeters with a length-to-diameter ratio as high as 10${}^{8}:1$ using techniques such as in situ gas-flow focusing, in computer simulations one typically proceeds by studying trends and saturation of their mechanical properties with respect to CNT length, which can be progressively incremented up to hundreds of nanometers. In this respect, mesoscale models can bridge the gap between molecular dynamics simulations and experimental scales. At the experimental level, an effective route that can be followed to create links in CNT bundles is to use carbon-ion beam irradiation with different irradiation intensities along the bundle by tuning the ion beam. An alternative approach to achieve this goal is by annealing in order to induce the migration of the interstitial linkers and modify their distribution. Furthermore, we verified that two novel potentials such as ReboScr2 and DRIP still show some shortcomings in the description of the mechanical properties of CNT bundles with carbon linkers.

## Data Availability

The data supporting the findings of this study are available within the article.
